# Sorghum-peanut intercropping under salt stress mediates rhizosphere microbial community shaping in sorghum by affecting soil sugar metabolism pathways

**DOI:** 10.3389/fmicb.2025.1589415

**Published:** 2025-05-01

**Authors:** Xia Shao, Chunmei Yang, Yuxuan Chen, Chang Liu, Chunjuan Liu, Xiaolong Shi, Yufei Zhou

**Affiliations:** College of Agronomy, Shenyang Agricultural University, Shenyang, China

**Keywords:** soil salinization, rhizosphere microenvironment, metagenome, soil metabolites, energy metabolism

## Abstract

Soil salinization is a substantial impediment to agricultural production, and investigating sustainable mitigation measures is essential for addressing food security. We conducted a two-year pot experiment to investigate the shaping mechanism of sorghum rhizosphere microbial community in sorghum-peanut intercropping system under salt stress. The experiment comprised four treatments: sole-cropped sorghum under normal soil conditions (NSS), intercropped sorghum under normal soil conditions (NIS), sole-cropped sorghum under salt-stress conditions (SSS), and intercropped sorghum under salt-stress conditions (SIS). The sorghum rhizosphere soil metabolites were examined using GC–MS, and the rhizosphere microbial community was characterized through metabolome sequencing. We identified 123 metabolites across treatments, with significant differences between normal and salt-stress soil conditions. The major metabolite classes included carbohydrates, alcohols, and acids. Key carbohydrates, including fructose and sucrose, were significantly reduced in the SIS than in SSS, NSS, and NIS treatments. Metabolic pathway analyses revealed that these differences were primarily associated with “Fructose and mannose metabolism,” “Starch and sucrose metabolism” and “ABC transporter.” Metabolome analyses revealed significant differences in microbial community structure across diverse soil conditions and cropping patterns. At phylum level, Proteobacteria, Gemmatimonadetes, and Verrucomicrobia predominated, with their relative abundance experiencing substantial changes under salt stress. SIS facilitated the enrichment of specific genera (*Rhodanobacter*), which were associated with soil health and stress tolerance. Additionally, the responses of rare microbial taxa to salt stress and intercropping varied, with specific rare microbial taxa (*Rhizopus*) exhibiting relative abundance under salt stress. Correlation analysis of metabolites and microbial taxa revealed that certain carbohydrates were significantly positively correlated with specific microbial phyla (Cyanobacteria and Nitrospirae) while demonstrating a significant negative correlation with Planctomycetota and Bacteroidota. These correlations indicate that sorghum intercropped with peanuts can promote the enrichment of microbial taxa under salt stress, thereby enhancing soil metabolic functions and stress tolerance by optimizing the rhizosphere microbial community. This study reveals the mechanism through which sorghum-peanut intercropping under salt stress influences the composition of sorghum’s rhizosphere microbial community by modulating soil sugar metabolism pathways. This finding provides a new perspective on sustainable agricultural practices in saline soils and emphasizes the pivotal role of plant-metabolite-microbe interactions in abiotic stress mitigation.

## 1 Introduction

Soil salinization is a serious environmental problem encountered in agriculture globally. Excessive salt stress due to soil salinization severely inhibits crop growth and development, jeopardizing crop production and ecological security (Perri et al., 2022; Zhao et al., 2021). Due to rising sea levels from global climate change and the exacerbation of imprudent irrigation practices, soil salinization is worsening significantly (Hopmans et al., 2021). Approximately 20% of the global irrigated land is affected by salinization, and this percentage continues to increase (Ma and Tashpolat, 2023). Considering the diminishing availability of arable land, the efficient utilization of salinized land has emerged as a pressing issue for agricultural production. Compared to the expensive renovation of salinized land, the choice of appropriate crops and the enhancement of cultivation practices may represent a more economical and practical approach.

Salinized soils are usually only conducive to the cultivation of salt-tolerant crops; however, long-term sole cropping might cause the degradation of soil properties and an imbalance in microbial diversity. Therefore, optimizing the cropping pattern of salinized land is essential. Intercropping, as a conventional and effective agricultural practice, can significantly enhance the structure and function of soil microbial communities by increasing biodiversity (Sun et al., 2024). Intercropping systems have obvious advantages over sole cropping in improving the soil microenvironment (including microbial community structure and soil nutrient cycling), mitigating disease incidence, and increasing crop nutrient resource utilization (Chamkhi et al., 2022; Li et al., 2014; Madembo et al., 2020). Intercropping in the peanut-cotton system significantly improved carbon cycling, nutrient supply, and microbial growth efficiency by optimizing soil microbial communities, thereby serving as an effective method to enhance the multifunctionality of soil ecosystems (Zhang et al., 2025). Corn-soybean intercropping significantly affected the bioavailability of soil phosphorus in the root zone by changing the microbial community structure (Gu et al., 2023). These studies demonstrated that ecological services facilitated by intercropping systems are closely related to the optimization of soil microbial community structure. Appropriate intercropping combinations create optimal habitats for microbial colonization and community structure optimization through interspecific interactions, which is essential for sustaining the multifunctionality of agroecosystems (Jiang et al., 2024b).

The rhizosphere soil is the core area for nutrient transformation and microbial activity and is the most critical area for plant growth (Song et al., 2020). Plant–soil-microbe rhizosphere interactions occur dynamically in the “black microzone” below the ground and are difficult to observe directly (Zhuang et al., 2024). Rhizosphere microorganisms play a decisive role in plant health and productivity by regulating various mechanisms of plant growth (Lin et al., 2021). Rhizosphere soil microbial communities are extremely diverse and complex and are significantly influenced by the rhizosphere chemical environment, especially metabolites (Solomon et al., 2024). Rhizosphere metabolites primarily originate from plant root exudates and soil microorganisms (Cheng et al., 2022), which act as chemical signals for rhizosphere interactions and are crucial for plant growth and development, disease suppression, environmental tolerance, and soil health maintenance (Zhuang et al., 2024). A previous study demonstrated that rhizosphere exudates are affected by microorganisms and drive plant responses to environmental adaptations (Huen et al., 2017). At the root-soil interface, key saccharides and organic metabolites derived from carbohydrate metabolism not only function as substrates for energy transduction to counteract environmental stress, but also mediate osmotic adjustment through compatible solute accumulation, thereby preserving homeostatic equilibrium under stress conditions (Keunen et al., 2013). Moreover, organic acids in rhizosphere exudates are recognized as key drivers of microbial recruitment and proliferation within the rhizosphere (Bargaz et al., 2017). They influence the structure of the rhizosphere microbiome by promoting the growth of specific microbial populations while inhibiting the development of other microbial populations, thereby facilitating microbial activity and improving nutrient conversion (Wang et al., 2023). Furthermore, rhizosphere microbes can enhance plant growth and improve soil nutrient levels by secreting phytohormones (growth hormones, cytokinins, and gibberellins) (Wood et al., 2016). Consequently, interactions between plant rhizosphere microbes and metabolites maintain the stability of microbial community structure and provide an important medium for plant–soil interactions.

Sorghum (*Sorghum bicolor* L.) is suitable for cultivation in marginal lands as a “hardcore crop” that is tolerant to salinity, drought, and barrenness (Huang, 2018). The peanut (*Arachis hypogaea* L.) exhibits moderate salt tolerance (Chakraborty et al., 2016). Both possess important value in the development and utilization of saline soils. Our previous studies have demonstrated that sorghum-peanut intercropping under salt stress can effectively change soil properties and microbial community structure (Shi et al., 2023); however, the precise modification of soil microbial community and metabolic profiles in the sorghum rhizosphere is unclear. Consequently, based on the sorghum-peanut intercropping system, we evaluated the metabolic profile and microbial metagenomic information of sorghum rhizosphere soil under different soil conditions to investigate the following: (1) Compared with solo-cropped sorghum, what specific changes occurred in the rhizosphere soil metabolites and microorganisms of intercropped sorghum under salt stress? (2) Do root metabolites play a dominant role in microbial community shaping?

## 2 Materials and methods

### 2.1 Site description and experimental design

The 2-year pot experiment was performed at the experimental site of Shenyang Agricultural University (41°82 N, 123°56E) between 2020 and 2021. The experimental site has a temperate continental climate with temperatures ranging from −36 to 40°C and an average annual rainfall of 710 mm. The experimental soil comprised farmland arable soil (brown loam) at the experiment station. The physical and chemical properties of the experimental soil was presented in Supplementary Table S1. Before sowing, the soil salinity concentration was adjusted to 2.5 g (NaCl) kg^−1^ to simulate moderate soil salinity levels in the field (S, 0.25% NaCl), while the untreated soil served as a control (N, 0.00% NaCl). The soil (90 kg per pot) was subsequently placed in a black cylindrical polyvinyl chloride (PVC) potting (equipment 100 cm high and 60 cm in diameter), and the same dose of 0.003 g N kg^−1^ soil^−1^ (urea), 0.05 g P_2_O_5_ kg^−1^ soil^−1^ (calcium superphosphate) and 0.08 g K_2_O kg^−1^ soil^−1^ (potassium sulfate) were applied to each pot as basal fertilizer. The soil was incubated under natural conditions for approximately 30 days to ensure relative stabilization of the soil microbial community before sowing.

The experiment was performed with the sorghum variety “Liaoza 15” and the peanut variety “Huayu 25.” Four treatments were established: (1) sole-cropped sorghum under normal soil conditions (NSS); (2) intercropped sorghum under normal soil conditions (NIS); (3) sole-cropped sorghum under salt-stress conditions (SSS); (4) intercropped sorghum under salt-stress conditions (SIS). Peanut and sorghum were cultivated using identical PVC potting equipment. In the intercropping treatment group, the roots of peanut and sorghum did not establish barriers, allowing for normal interactions. In the solo-cropping treatment group, PVC partitions were installed between sorghum and peanut to prevent root interactions and the interchange of root exudates. Each pot contained three peanut plants and two sorghum plants, with a spacing of 15 cm between peanut plants, 20 cm between sorghum plants, and 40 cm between rows, and each treatment was replicated thrice. All treatments were subjected to the same field management during the experiment.

### 2.2 Rhizosphere soil sample collection

Rhizosphere soil was collected 60 days after sorghum germination in 2021. Before collection, the surface soil (0–5 cm) and other impurities (plant debris) surrounding the sorghum root system were carefully eliminated, and the soil adhering to the roots was gathered. The rhizosphere soil samples from the same treatment group were combined to obtain a mixed sample. After passing through a 2 mm sieve, the rhizosphere soil was aliquoted into 10 mL sterilized centrifuge tubes and stored in a −80°C refrigerator for the preparation of soil metabolites and metagenome extraction.

### 2.3 Metabolite detection and data analysis

To delineate the rhizosphere soil metabolite profiles, the collected soil rhizosphere samples were vacuum freeze-dried and analyzed using GC–MS metabolomic assay. The extraction and detection process were briefly summarized below: First, 2 g of soil sample were introduced into a centrifuge tube, to which 10 mL of pre-cooled extract [methanol: isopropanol: water (3:3:2 ratio)] was added, shaken for 3 min, and subsequently homogenized in an ice bath with ultrasonic irradiation for 20 min, followed by derivatization by centrifugation for 3 min at 12,000 r·min^−1^ at 4°C. The extract was subsequently employed for the derivatization of the soil sample. The resultant supernatant was gathered for injection analysis. An Agilent 7890B gas chromatography-7000D mass spectrometer (GC–MS) was used for GC–MS analysis of secondary metabolites in soil samples.

After acquiring the mass spectral data, the GC–MS analytical data was initially deconvoluted in batch mode utilizing Automated Mass Spectrum Deconvolution and Identification System (AMDIS) software. After analyzing the mass spectrum data detected from each sample against the self-constructed MetWare database (S_TMS_MWGC), the metabolite characteristics were ascertained based on the retention index and description of relevant scientific literature. We utilized MassHunter software to match quantitation ions to an in-house library of confirmatory standards for metabolite identification and metabolite quantification based on peak area integration. The R function prcomp (http://www.r-project.org) was used for statistical analysis of metabolites. Principal component analysis (PCA) was performed, and metabolite data were log-transformed and normalized before PCA. Subsequently, orthogonal partial least squares discriminant analysis (OPLS-DA) was employed to validate the PCA model and initially identify the differentially expressed metabolites (DEMs). Fold change (FC) ≥ 1.3 and *p*-value ≤ 0.05 were employed to identify significantly regulated metabolites between groups. Metabolite annotation was performed using the Kyoto encyclopedia of genes and genomes (KEGG).

### 2.4 Rhizosphere soil metagenome sequencing and data analysis

A 3 g soil sample was accurately weighed, and the genomic DNA was extracted using the DNeasy PowerMax Soil Kit (Qiagen, Hilden, Germany). After DNA was tested for purity and integrity using 1% agarose gel electrophoresis (AGE), quantification of soil DNA concentration was performed using the Qubit^®^dsDNA detection kit in a fluorometer. Qualified DNA samples were subjected to end repair, addition of sequencing splices, purification, PCR amplification, and other steps that were sufficient for sequencing library construction. After library construction was completed, library inspection was performed, and qualified samples were subjected to pair-end sequencing on the Illumina HiSeq4000 PE150 platform at Metwel Biotech (Wuhan, China).

Upon acquiring the raw sequencing data (Raw Data), we initially performed quality control and host filtering to eliminate low-quality base Reads and overlapping Reads and subsequently filtered out highly specific sequences potentially of host origin by referencing the genomes of sorghum and peanut using the Bowtie2 software, thereby obtaining the valid data (Clean Data) for subsequent analyses. Metagenome assembly was performed using MEGAHIT software with default settings, and the assembled Scaffolds were broken from the N junctions to obtain N-free sequence fragments termed Scaftigs. Open reading frame (ORF) prediction was performed from assembled Scaftigs (≥500 bp) using MetaGeneMark with default parameters. The CD-HIT software (Fu et al., 2012) was used to remove redundancy from ORFs (≥100 nt) to generate an initial non-redundant gene catalog (nrGC). The Clean Data of each sample were compared to the initial nrGC using Bowtie2, and the number of Reads on which the gene was compared in each sample was calculated. Genes with Reads ≤ 2 in individual samples were excluded to obtain non-redundant genes (Unigenes) for subsequent analysis.

Unigenes were aligned with microbial sequences drawn from the NCBI nr database using DIAMOND software (Buchfink et al., 2015) to acquire Unigenes phylogenetic information. The LCA algorithm, which is based on MEGAN software, was used to ascertain microbial taxa annotation information. The taxonomic annotation and abundance data of the metagenome were produced based on LCA annotation results and the gene abundance table.

### 2.5 Statistical analysis

Significant differences in differential metabolites were analyzed using one-way analysis of variance (*p <* 0.05). Spearman correlation heatmap analysis was used to characterize the correlation between differential metabolites and soil microorganisms. Calculations and plots were performed using the OmicStudio cloud platform (https://www.omicstudio.cn/tool/62).

## 3 Results

### 3.1 Rhizosphere soil metabolite information and differences

**Figure 1:**
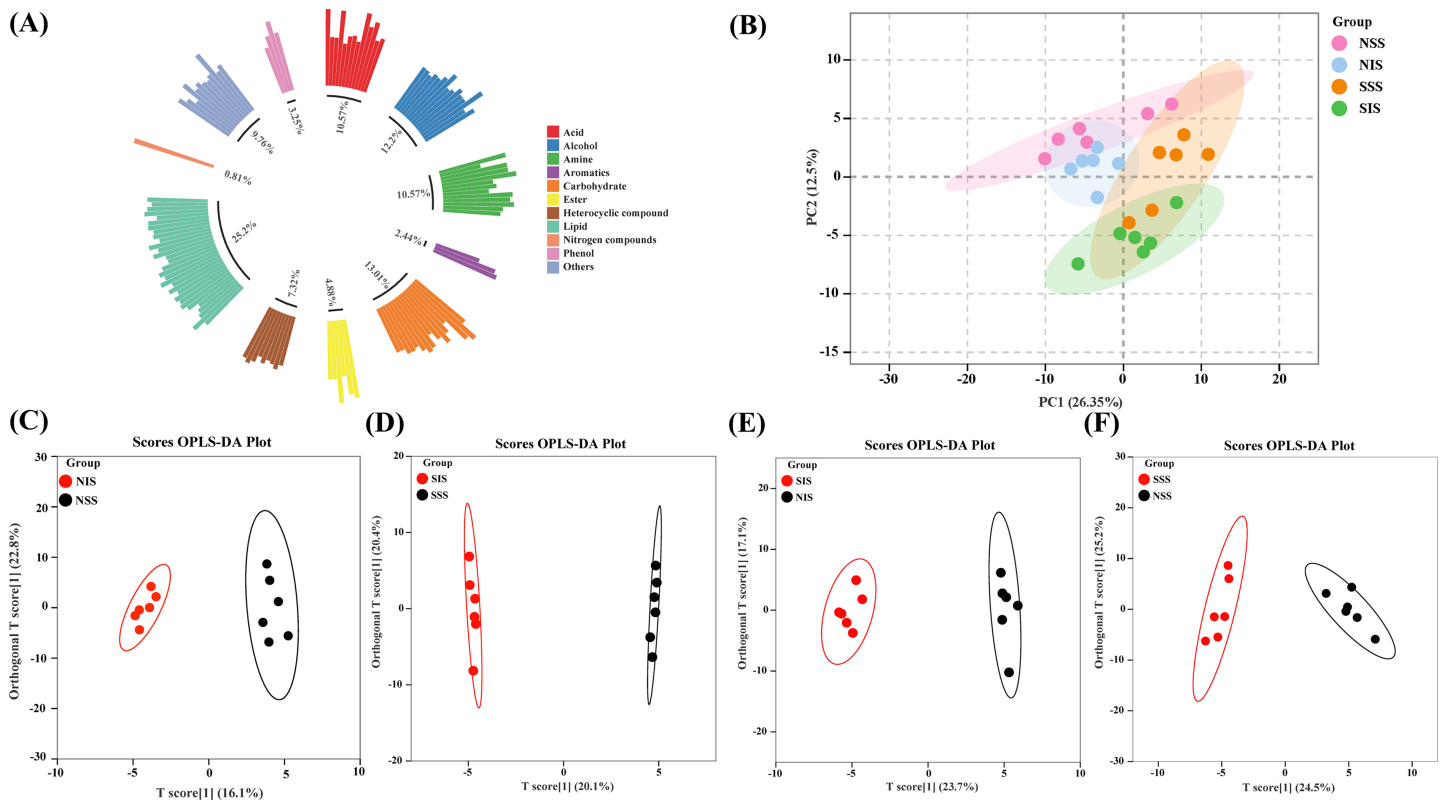
Rhizosphere soil metabolite profile analysis and assessment of intergroup differences. **(A)** Rhizosphere soil metabolite identification and classification. **(B)** PCA of metabolic profiles of samples. **(C–F)** OPLS-DA analysis of metabolic profiles in the NIS vs. NSS, SIS vs. SSS, SIS vs. NIS, and SSS vs. NSS comparison groups, respectively.

Based on the GC–MS non-targeted metabolome assay, we detected 123 metabolites in the four treatments of NSS, NIS, SSS, and SIS (Supplementary Table S1). The dataset comprises 31 lipids, 16 carbohydrates, 15 alcohols, 13 acids, 13 amines, 9 heterocyclic compounds, 6 esters, 4 phenols, 3 aromatics, 1 nitrogen compound, and 12 others (Figure 1A).

PCA revealed that PC1 and PC2 collectively accounted for 38.85% of the variation in metabolites across samples. The metabolites from each sample under normal soil conditions (N) and salt-stress soil conditions (S) were separated along PC1 (26.35%); however, the metabolic profile differences between cropping patterns, sole cropping (SS), and intercropping (IS) were non-significant, with PC2 accounting for merely 12.49% of the variance (Figure 1B). The OPLS-DA revealed that the six biological replicates within the same sample in the NIS vs. NSS, SIS vs. SSS, SIS vs. NIS, and SSS vs. NSS comparison groups were clustered into one group. Furthermore, a significant distinction existed between the two samples, indicating that there were significant differences in metabolites of soil samples across different treatments (Figures 1C–F).

### 3.2 Screening of DEMs

**Figure 2:**
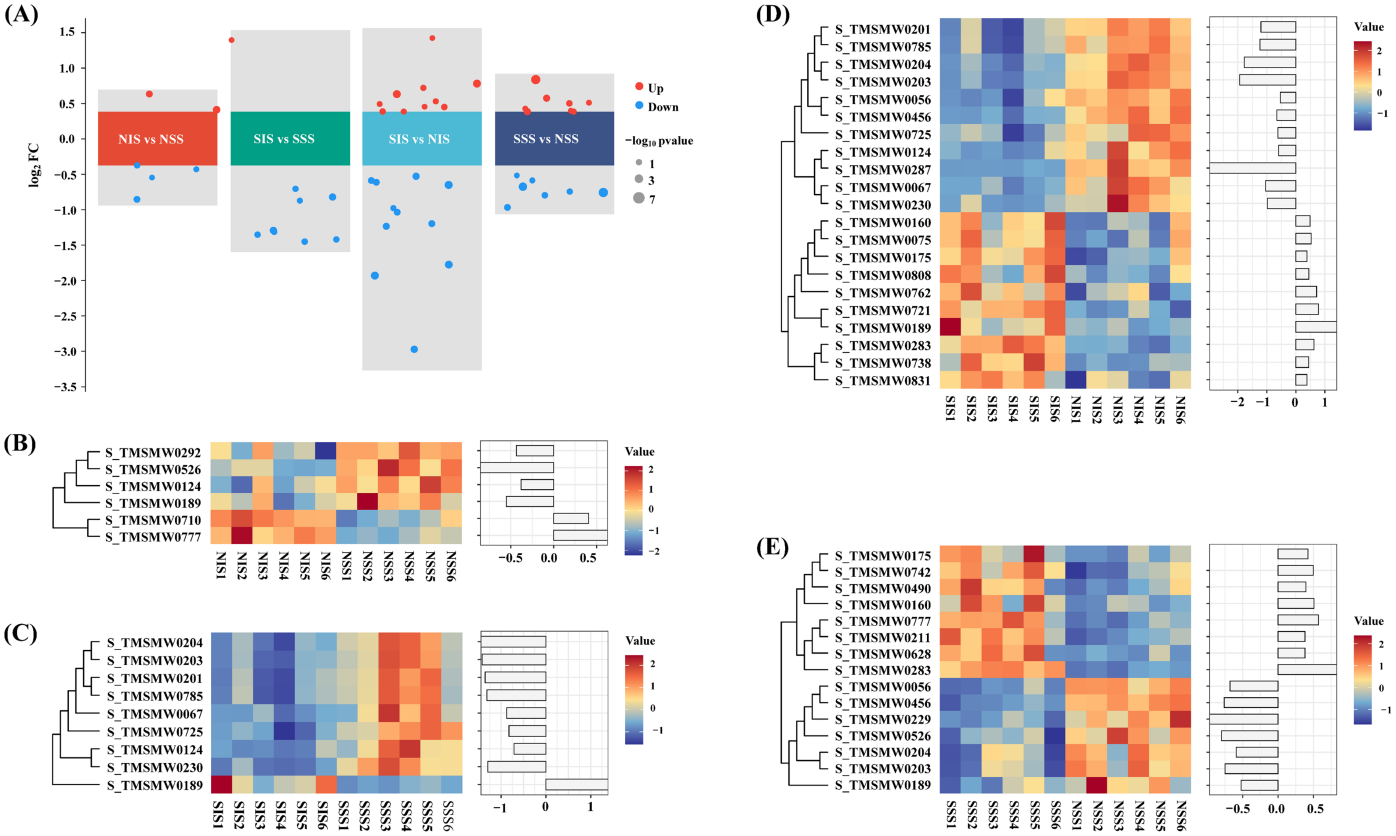
Differential metabolite screening and differential analysis. **(A)** The volcano plot of differential metabolite in each comparison group. **(B–E)** The heatmap of metabolite abundance differences in the NIS vs. NSS, SIS vs. SSS, SIS vs. NIS, and SSS vs. NSS comparison groups, respectively.

The screening criteria for identifying DEMs in each comparison group were set at FC ≥ 1.3 and *p* < 0.05 based on the relative abundance information of metabolites in each sample. As illustrated in Figure 2A, 6 DEMs (2 up-and 4 down-regulated) were screened in the NIS vs. NSS comparison group, 15 DEMs (8 up-and 7 down-regulated) were screened in the SSS vs. NSS comparison group, 9 DEMs (1 up-and 8 down-regulated) were screened in the SIS vs. SSS comparison group, and 21 DEMs (10 up-and 11 down-regulated) were screened in the SIS vs. NIS comparison group. In NIS vs. NSS, SIS vs. SSS, and SIS vs. NIS comparison groups, S_TMSMW0189(2-(4’-Methoxyphenyl)-2-(3’-methyl-4’methoxyphenyl)propane) was significantly up-regulated (Figures 2B–D). In SIS vs. SSS, SIS vs. NIS, and SSS vs. NSS comparison groups, S_TMSMW0203 (D(+)-Talose) and S_TMSMW0204 (D-Allose 2) were significantly down-regulated (Figures 2C–E). In SSS vs. NSS and SIS vs. NIS comparison groups, S_TMSMW0456 (Rhamnose) and S_TMSMW0056 (d-Arabinose 2) were significantly down-regulated; however, S_TMSMW0283 (1,6-anhydro-.beta.-D-Glucopyranose), S_TMSMW0175 (1-iodo-Dodecane) and S_TMSMW0160 (2-(4′-Hydroxyphenyl)-2-(4′-methoxyphenyl)propane) were significantly up-regulated (Figures 2D,E). DEMs were primarily clustered into carbohydrates, alcohols and acids (Supplementary Figure S1). All metabolite numbers are indicated in Supplementary Table S2.

### 3.3 Functional annotation of DEMs

**Figure 3:**
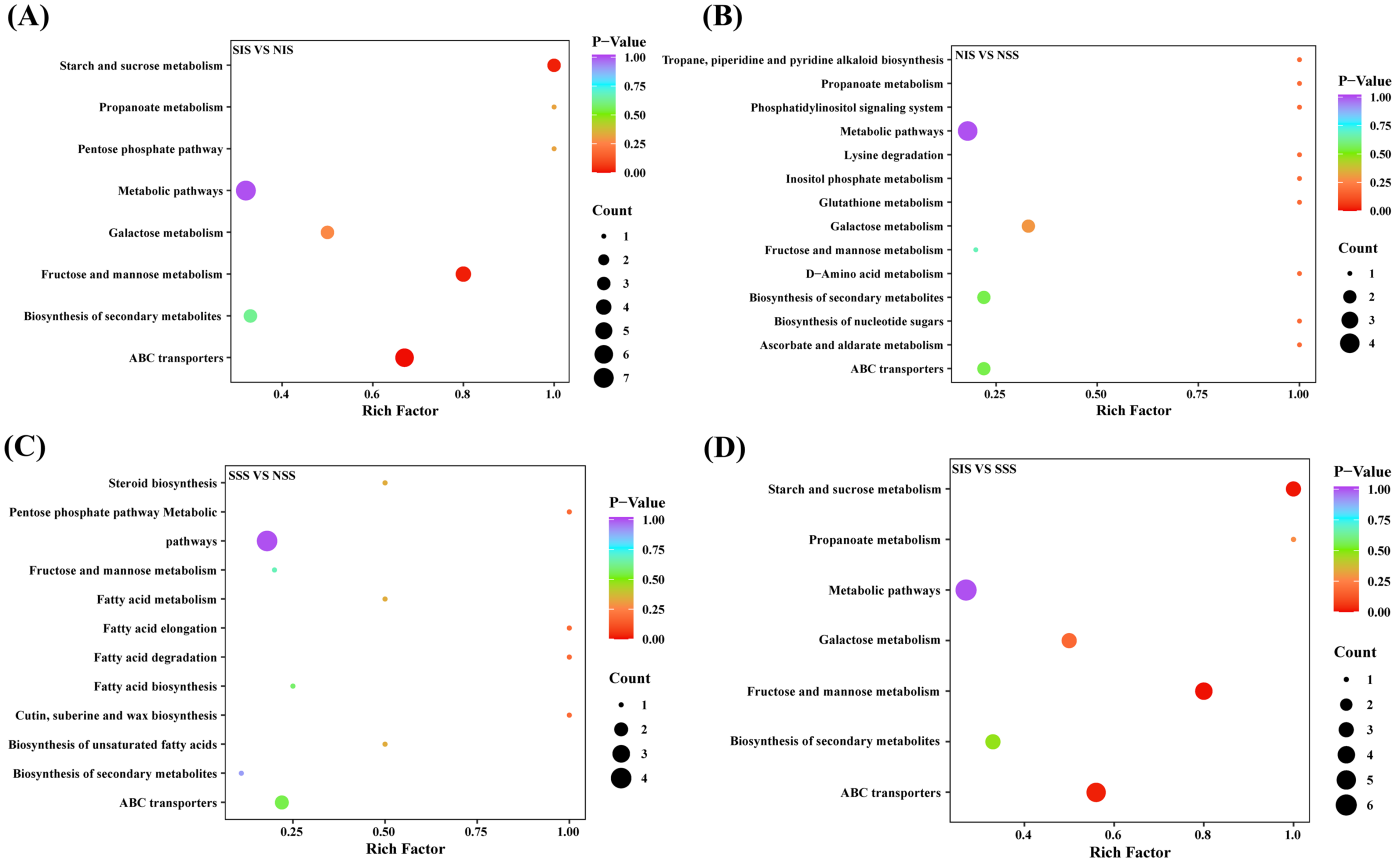
Top metabolic pathways in comparison groups. **(A–D)** The bubble map of KEGG enrichment of differential metabolites in the SIS vs. NIS, NIS vs. NSS, SSS vs. NSS, and SIS vs. SSS comparison groups, respectively. The color from purple to red indicates that the *p*-value decreases; the larger the dot, the more metabolites are enriched on the pathway.

Figure 3 depicts that the metabolic pathways significantly enriched in the four treatments comprised “Starch and sucrose metabolism,” “Fructose and mannose metabolism,” “Galactose metabolism,” “Biosynthesis of secondary metabolites,” “Metabolic metabolism” and “ABC transporters.” No significantly enriched metabolic pathways were observed in the NIS vs. NSS and SSS vs. NSS comparison groups (Figures 3B,C). In SIS vs. SSS and SIS vs. NIS comparison groups, DEMs were significantly enriched in “Starch and sucrose metabolism,” “Fructose and mannose metabolism,” and “ABC transporters” metabolic pathways (Figures 3A,D). S_TMSMW0201, S_TMSMW0230, and S_TMSMW0785 were significantly enriched in the “Starch and sucrose metabolism” metabolic pathway; S_TMSMW0067, S_TMSMW0201, S_TMSMW0230, and S_TMSMW0785 were significantly enriched in the “Fructose and mannose metabolism” metabolic pathway; S_TMSMW0067, S_TMSMW0201, S_TMSMW0204, S_TMSMW0230, and S_TMSMW0785 were significantly enriched in the “ABC transporters” metabolic pathway. Additionally, the metabolites mentioned above, which belong to sugars and alcohols, were down-regulated in each pathway (Supplementary Table S3). The DEMs in SIS rhizosphere soil were more involved in “Starch and sucrose metabolism,” while the DEMs in SSS rhizosphere soil were more related to “Fatty acid elongation” and “Fatty acid degradation” metabolic pathways.

### 3.4 Structure and composition of rhizosphere soil microbial community

**Figure 4:**
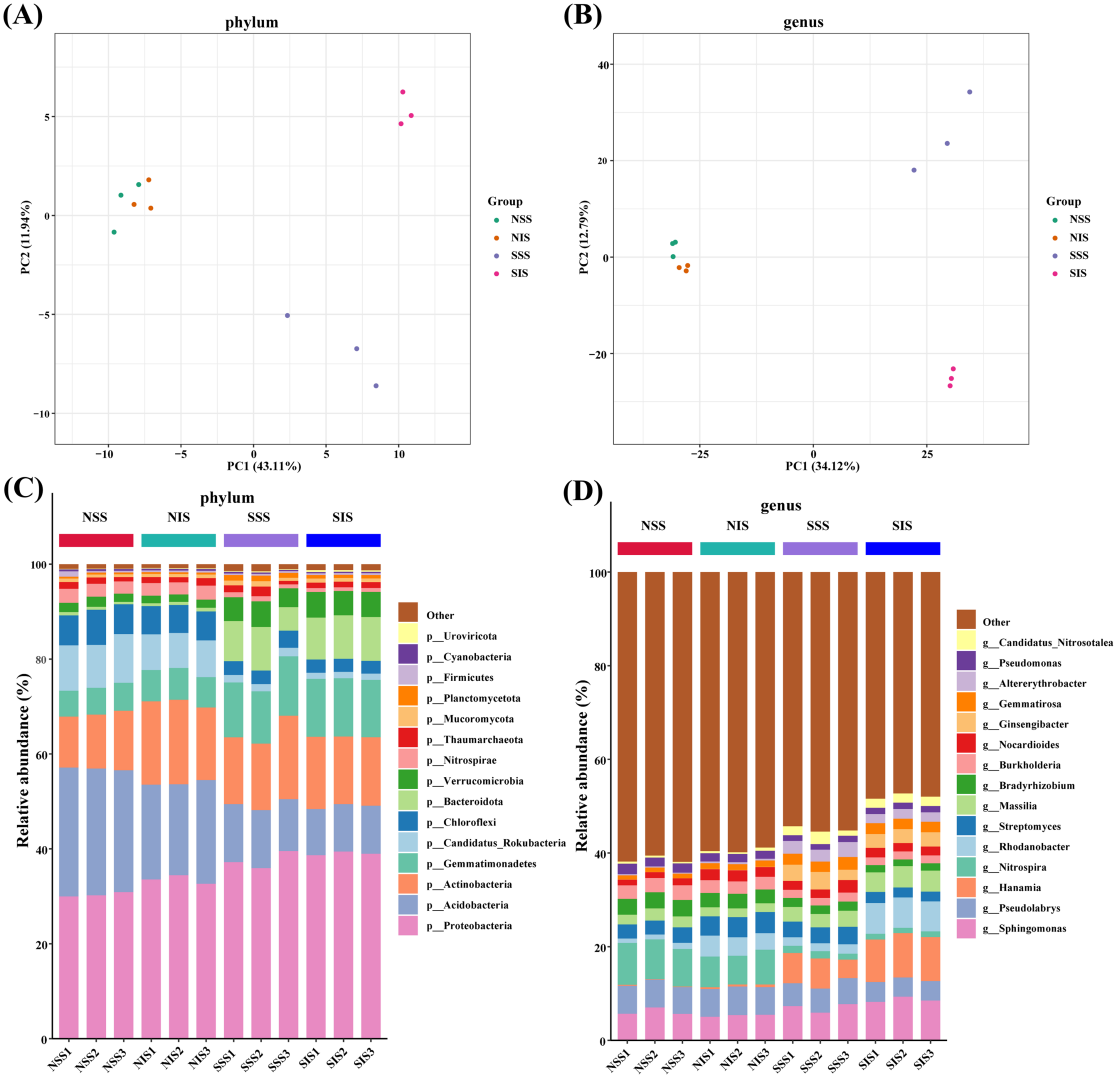
PCA and relative abundance information of sorghum rhizosphere soil microbial community at the phylum and genus levels. **(A,B)** The PCA of the sorghum rhizosphere soil microbial community at the phylum and genus levels, respectively. **(C,D)** The relative abundance information of sorghum rhizosphere soil microbial community at the phylum and genus levels, respectively.

Core and pan genome analysis revealed fundamental genomic characteristics across studied strains. The number of shared genes (core genes) decreased and the number of total genes (pan genes) increased in all samples with increasing random samples number, revealed that the metagenome sequencing sample size was adequate, and the data met the criteria for subsequent analysis (Supplementary Figure S2). Based on PCA and top 15 species composition relative abundance information. Significant differences were observed in microbial community structure at the phylum level. Under normal soil conditions (N) and salt-stressed soil conditions (S), the microbial community was separated along PC1 (43.11%); however, there was a non-significant difference between solo cropping (SS) and intercropping (IS) (Figure 4A). At the phylum level, Proteobacteria, Acidobacteria, Actinobacteria, Gemmatimonadetes, Candidatus_Rokubacteria, Chloroflexi, Bacteroidota, and Verrucomicrobia were the dominant microbial taxa in sorghum rhizosphere soil (Figure 4C). The relative abundances of Proteobacteria, Gemmatimonadetes, Bacteroidota, and Verrucomicrobia were significantly higher in salt-stress soil than in normal soil. The relative abundances of Cyanobacteria, Nitrospirae, Candidatus_Rokubacteria, Acidobacteria, and Chloroflexi were significantly higher in normal soil than in salt-stress soil (Supplementary Figure S4A). PCA at the genus level revealed significant differences in microbial community structure among treatments, with a microbial community of each sample under normal and salt-stressed soil conditions separated along PC1 (34.12%), and sole cropping and intercropping were separated along PCA2 (12.79%) (Figure 4B). At the genus level, *Sphingomonas, Pseudolabrys, Hanamia,* and *Nitrospira* exhibited the highest relative abundance across the samples (Figure 4D). The relative abundances of *Hanamia, Rhodanobacter, Massilia, Ginsengibacter, Gemmatirosa, Altererythrobacter, Sphingomonas,* and *Candidatus_Nitrosotalea* were significantly higher in salt-stressed soil than in normal soil, the relative abundances of *Nitrospira* and *Bradyrhizobium* were significantly higher in normal soil than in salt-stressed soil. The relative abundances of *Rhodanobacter* were significantly higher in intercropping than in sole cropping (Supplementary Figure S4B).

### 3.5 Differential analysis of rhizosphere soil rare microbial taxa

**Figure 5:**
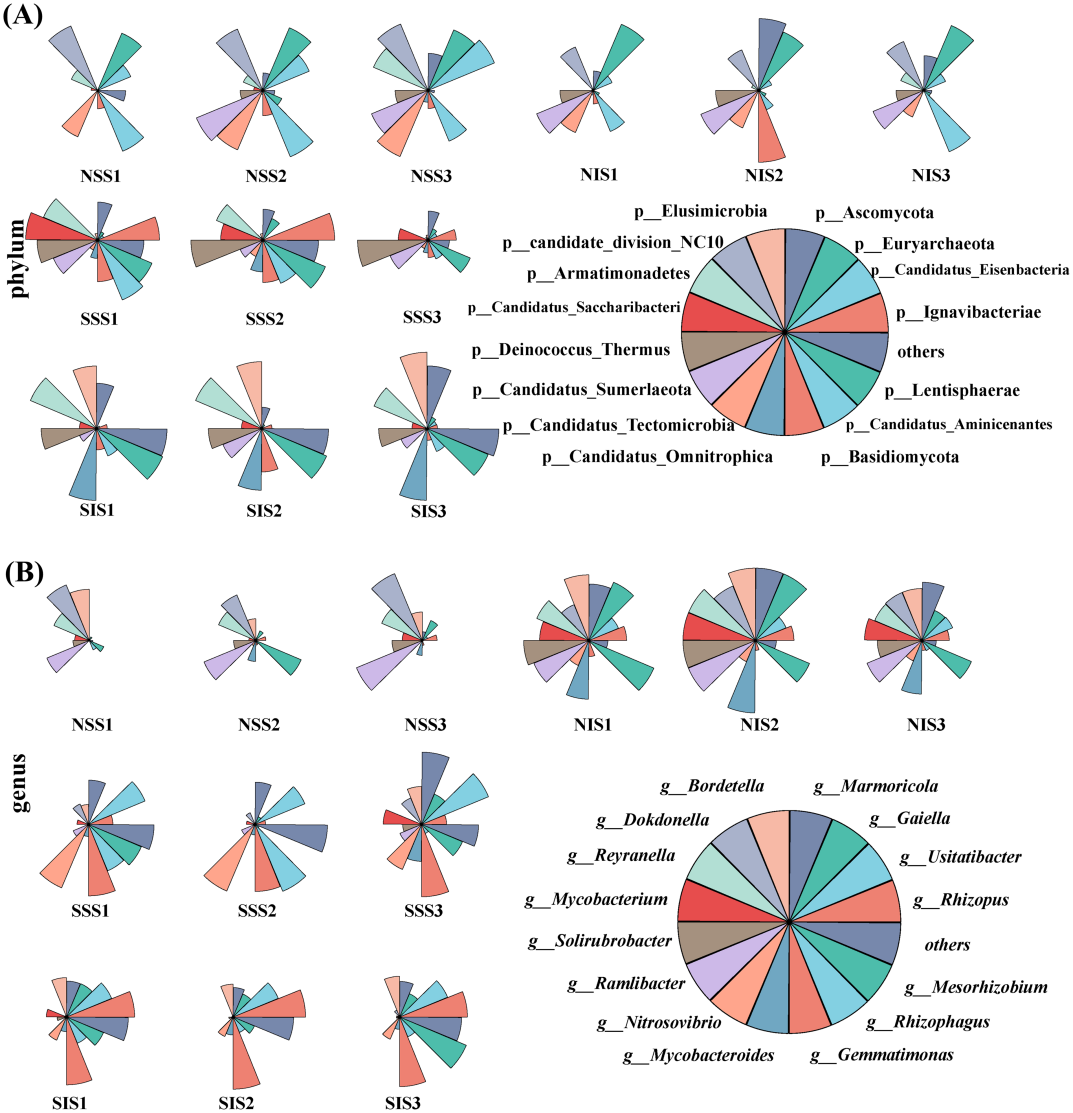
Relative abundance of rare microbial taxa in sorghum rhizosphere soil. **(A,B)** The relative abundance of rare microbial taxa at the phylum and genus levels, respectively.

In addition to examining the response of predominant microbial taxa with higher relative abundance to treatment (soil conditions, cropping patterns), we analyzed the variations in the relative abundance of rare microbial taxa (those with relative abundance < 0.1%) across the samples. At the phylum level, the relative abundances of Elusimicrobia, Ignavibacteriae, Candidatus_Omnitrophica, and Lentisphaerae were significantly higher in salt-stress soil than in normal soil. The relative abundances of Euryarchaeota, Candidate_division_NC10, and Candidatus_Tectomicrobia were significantly higher in normal soil than in salt-stress soil (Figure 5A). At the genus level, the relative abundances of *Usitatibacter* and *Gemmatimonas* were significantly higher in salt-stress soil than in normal soil. The relative abundances of *Reyranella*, *Dokdonella*, and *Ramlibacter* were significantly higher in normal soil than in salt-stress soil. The relative abundance of *Rhizopus* was significantly higher in intercropping than in sole cropping (Figure 5B).

### 3.6 Information on microbial community functions

**Figure 6:**
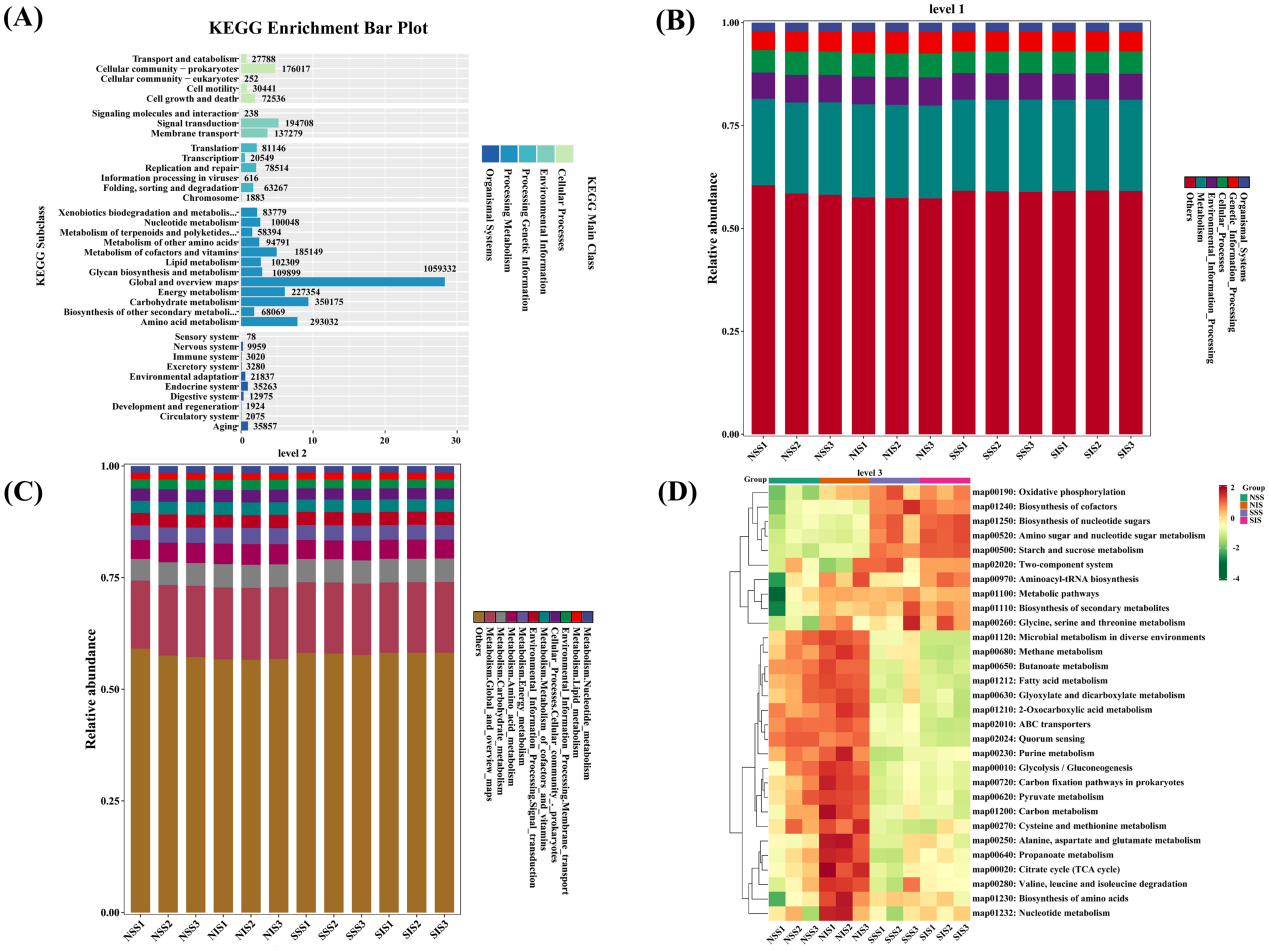
KEGG functional annotation of sorghum rhizosphere soil microbiome. **(A)** The gene number involved in the annotation of KEGG pathways. **(B–D)** The abundance of KEGG pathways in levels 1, 2, and 3, respectively.

As depicted in Figure 6, the Unigenes in each process can be classified into five categories within the level 1 metabolic pathway, with “Metabolism” exhibiting the highest gene annotation count. The relative abundances of “Environmental Information Processing,” “Cellular Processes,” and “Organismal Systems” were significantly higher in NIS and NSS than in SIS and SSS; however, “Cellular Processes” and “Genetic Information Processing” exhibited significantly higher relative abundance in SIS than in SSS (Figure 6B). The level 2 metabolic pathways with the highest number of enriched genes in “Global and overview maps,” “Carbohydrate metabolism,” “Amino acid metabolism,” “Energy metabolism,” and “Signal transduction” (Figure 6C). Under salt stress, the level 3 metabolic pathway of KEGG was primarily enriched in “Oxidative phosphorylation,” “Biosynthesis of cofactors,” “Starch and sucrose metabolism,” “Biosynthesis of nucleotide sugars,” and “Amino sugar and nucleotide sugar metabolism.” Compared to sole-cropping treatments, intercropping treatments increased the relative abundances of “Oxidative phosphorylation,” “Starch and sucrose metabolism,” and “Glycine, serine, and threonine metabolism” in sorghum rhizosphere soil (Figure 6D).

### 3.7 Combined analysis of microbes and metabolites

**Figure 7:**
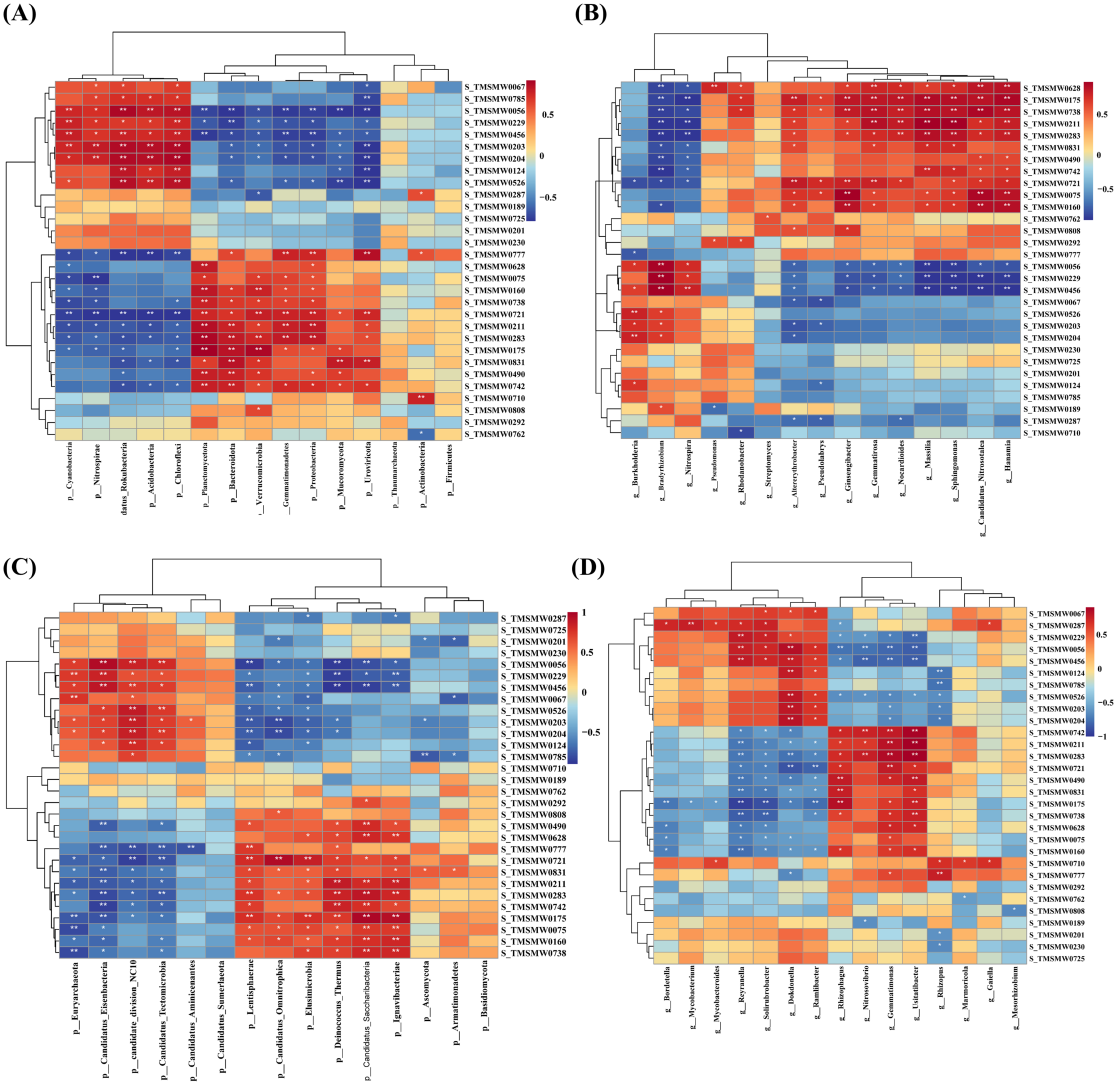
Correlation analysis between microbial taxa and metabolites. **(A,B)** The correlation of dominant microbial taxa and metabolisms at the phylum and genus levels. **(C,D)** The correlation of rare microbial taxa and metabolisms at the phylum and genus levels, respectively. 0.01 < *p* < 0.05*, 0.001 < *p* < 0.01**.

Based on the screened differential metabolites and microbial taxa, the relative abundance of the top 15 dominant microbial taxa was selected for association analysis with differential metabolites. At the phylum level, Cyanobacteria, Nitrospinae, Candidatus_Rokubacteria, Acidobacteria, and Chloroflexi were significantly positively correlated with S_TMSMW0056, S_TMSMW0229 (D-(−)-Ribofuranose 1), S_TMSMW0456, S_TMSMW0203, and S_TMSMW0204. Candidatus_Rokubacteria, Acidobacteria, and Chloroflexi were positively correlated with S_TMSMW0124 (Propylene Glycol) and S_TMSMW0526 (9-Hexadecenoic Acid) but significantly negatively correlated with S_TMSMW0777 (4-hydroxy-Benzonitrile), S_TMSMW0721 (trans-O-Dithiane-4,5-diol), S_TMSMW0211 (n-Hexadecanoic Acid), and S_TMSMW0283. Conversely, Planctomycetota, Bacteroidota, Verrucomicrobia, Gemmatimonadetes, and Proteobacteria were significantly positively correlated with S_TMSMW0160, S_TMSMW0738 (1-iodo-Eicosane), S_TMSMW0721, S_TMSMW0211, S_TMSMW0283, S_TMSMW0175, and S_TMSMW0742 (Andrographolide), and significantly negatively correlated with S_TMSMW0056 and S_TMSMW0456 (Figure 7A). At the genus level, *Bradyrhizobium* and *Nitrospinae* were significantly positively correlated with S_TMSMW0056, S_TMSMW0229, and S_TMSMW0456. *Ginsengibacter, Gemmatirosa, Nocardioides, Massilia, Sphingomonas, Candidatus_Nitrosotalea,* and *Hanamia* were significantly negatively correlated with S_TMSMW0056, S_TMSMW0229, and S_TMSMW0456 (Figure 7B).

Correlation analyses between rare microbial taxa and metabolite revealed that Euryarchaeota, Candidatus_Eisenbacteria, Candidate_division_NC10, and Candidatus_Tectomicrobia were significantly positively associated with S_TMSMW0056, S_TMSMW0229, S_TMSMW0456, S_TMSMW0067, S_TMSMW0526, S_TMSMW0203, S_TMSMW0204, and S_TMSMW0124; Lentisphaerae, Elusimicrobia, and Deinococcus_Thermus were significantly positively associated with S_TMSMW0056, S_TMSMW0203, S_TMSMW0204, S_TMSMW0229, and S_TMSMW0456 (Figure 7C). At the genus level, *Reyranella, Solirubrobacter, Dokdonella,* and *Ramlibacter* were significantly positively correlated with S_TMSMW0056 and S_TMSMW0456. *Dokdonella* were significantly positively correlated with S_TMSMW0067, S_TMSMW0229, S_TMSMW0124, S_TMSMW0526, S_TMSMW0203, and S_TMSMW0204 (Figure 7D).

## 4 Discussion

In rhizosphere soil, plant root exudates and microbial metabolites constitute complex metabolic profiles. Particularly under stress conditions, specific changes in these metabolic profiles reflect the adaptive responses of plants and affect the structure and function of microbial communities. Herein, we investigated changes in microbial community structure and metabolic profile characteristics of sorghum rhizosphere soil subjected to salt stress in a sorghum-peanut intercropping system and investigated the interaction relationship between the microbiota and metabolites. The findings demonstrated that intercropping substantially influenced the sorghum rhizosphere microbial community structure by affecting soil sugar metabolism pathways, offering a potential protective mechanism for plant growth under salt stress.

### 4.1 Interactions between rhizosphere soil metabolites and microbial communities

Intercropping can substantially modify the composition and diversity of rhizosphere soil metabolites (Chen et al., 2024). This study revealed that the types and concentrations of rhizosphere soil metabolites in the sorghum-peanut intercropping system changed significantly under salt stress. Specifically, lipids were the most abundant, followed by carbohydrates, alcohols, amines, and acids (Figure 1A). Furthermore, the composition of root metabolites exhibited significant differences across treatments (Figures 1C–F). Specifically, significant consumption of sugar metabolites was observed in SIS vs. SSS, SIS vs. NIS, and SSS vs. NSS comparison groups (Figures 2C–E). KEGG enrichment analysis revealed that these sugar metabolites were significantly enriched in “Fructose and mannose metabolism” and “Starch and sucrose metabolism” (Supplementary Table S2). These results revealed that sorghum and associated rhizosphere microbes can maintain normal survival under salt stress by accelerating sugar metabolism processes. Sugar metabolism is essential in sorghum’s resistance to salt stress. Carbajal-Vázquez et al. (2022) revealed that sugars are important organic compounds associated with plant salt tolerance. Additionally, sugar metabolites, as vital energy sources, undergo substantial consumption during metabolic processes to provide primary energy for plants resisting salt stress, and serve as substrates to support the recruitment and remodeling of microbial communities.

Additionally, root-rhizosphere interactions in intercropping systems substantially influence the chemical composition and diversity of rhizosphere metabolites. For instance, intercropping with leguminous crops can modify the composition of rhizosphere metabolites in tea plants (Wang et al., 2023). Root-rhizosphere interactions in intercropping systems can enhance rhizosphere soil microbial diversity and the secretion of specific root exudates (Sun et al., 2024; Zhou et al., 2023). This study demonstrated that the substantial consumption of sugar metabolites in the sorghum rhizosphere soil after sorghum intercropped with peanut, this might explain that intercropping improves the metabolic activity of microbial community by accelerating the turnover of energy metabolism such as sugars, and consequently, exhibits greater microbial community structural diversity and activity under salt-stress conditions.

### 4.2 Effect of intercropping on rhizosphere microbial community structure

The rhizosphere microbiota is essential for plant stress tolerance (Saleem et al., 2019). Salt-tolerant rhizosphere bacteria can enhance plant adaptation to salt stress by producing abundant secondary metabolites (Abbas et al., 2019). Root exudates are a crucial medium connecting plant and soil microbes (Chai and Schachtman, 2022). The changes and positive impacts of intercropping on soil microbial communities were primarily attributed to plant root organic matter deposition and exudation of root metabolites (Chen et al., 2019). Herein, we demonstrated that salt stress significantly altered the rhizosphere soil microbial community structure and diversity of sorghum. Proteobacteria, Gemmatimonadetes, Bacteroidota, and Verrucomicrobia were the dominant phyla of the microbial communities, which is consistent with the findings of Bao et al. (2024). Furthermore, these microbial taxa have a higher relative abundance in intercropping rhizosphere soil than in sole cropping, indicating that intercropping enhanced the stability and resistance of the rhizosphere microenvironment by enhancing the microbial community structure. Proteobacteria is the main phylum in the rhizosphere of most plant species, capable of degrading aliphatic and aromatic compounds into simpler forms and enduring extreme conditions (Xu et al., 2022). Gemmatimonadetes are highly adaptable to high-salinity soils (Guan et al., 2021), and their metabolites can provide nutrients for plant growth and improve the ability of plants to resist environmental stress (Zhou et al., 2024). Many genera of *Mycobacterium* make positive contributions to plant health (disease control and resistance to multiple abiotic stresses) and growth development. For instance, the colonization of *Flavobacterium* could enhance the stress tolerance of plants by activating molecular pathways related to the phytohormone abscisic acid and improve disease resistance by inhibiting the proliferation of fungal pathogens (Seo et al., 2024).

The relative abundance of *Rhodanobacter* at the genus level was significantly higher in the intercropping treatment than in the sole cropping treatment under salt stress (Supplementary Figure S3B). As a member of the Proteobacteria, *Rhodanobacter* were chemoautotrophs bacteria, participates in Hg^0^ oxidation, nitrification and denitrification simultaneously (Huang et al., 2019). In addition, the composition and structure of rare microbial taxa in the rhizosphere soil changed significantly. The relative abundances of *Rhizopus* were significantly higher in the intercropping treatment than in the sole cropping treatment under salt stress (Figure 5B). These results indicated that intercropping affected the structure of dominant microbial taxa and significantly changed the composition of rare microbial taxa, further optimizing the rhizosphere microenvironment.

### 4.3 Correlation of rhizosphere soil metabolites with microbes

The proliferation of microbial taxa is closely associated with the variation of compounds in the rhizosphere environment. Metabolites released by the root system of the host plant were a key factor in driving the recruitment of specific microbial taxa (Parasar et al., 2024). Root metabolites are signaling molecules that can affect the soil microbiome, including promotion, inhibition, and expulsion (Jiang et al., 2024a). Furthermore, the metabolic activities of the rhizosphere soil microbiome are essential in shaping the rhizosphere chemical composition (Song et al., 2020). This study demonstrated that the contents of sugar metabolites (d-Arabinose 2, Rhamnose, D(+)-Talose, D-Allose 2, Fructose 2, Fructose 1, 1-Deoxy-d-arabitol, and Sucrose) in the rhizosphere soil were significantly lower in salt-stressed soil conditions than in normal soil conditions. Furthermore, significant consumption of energy substances, including sugars, was found in the rhizosphere soil of intercropping treatments under salt stress. This consumption of energy substances was closely associated with the remodeling of microbial community structure and recruitment of specific microbial taxa under stress conditions. The microbial-metabolite correlation analysis revealed that Bacteroidota, Verrucomicrobia, Gemmatimonadetes, *Massilia*, and *Sphingomonas* exhibited significant negative correlations with d-Arabinose 2 and Rhamnose, suggesting that the enrichment of these microbial taxa may require substantial consumption of sugars as energy substrates. Song et al. (2020) reported that microbial taxa in soil that positively correlated with sugar metabolites might release sugars under low-sugar conditions, while an increase in microbial taxa that negatively correlated with sugar metabolites may cause more severe sugar consumption. Research on plant-microbe interactions has revealed that *Sphingomonas* spp. confer salt stress mitigation in *Arabidopsis thaliana* seedlings through enzymatic degradation of reactive oxygen species (ROS) maintain redox homeostasis under saline conditions (Jiang et al., 2023). The co-inoculation of arbuscular mycorrhizal fungi (AMF) and plant growth-promoting *Massilia* strains demonstrates synergistic effects in enhancing salt tolerance in maize (Krishnamoorthy et al., 2016). Microbiome KEGG functional annotation revealed that the metabolic functions of the differential microbial taxa were predominantly enriched in carbohydrate, energy, and amino acid metabolism pathways (Figure 6D). As a result, complex and close relationships exist between rhizosphere soil metabolites and microbial communities. Under environmental stress conditions, plants recruit specific microbial taxa by regulating the composition of rhizosphere metabolites, thereby facilitating their involvement in specific metabolic processes in response to environmental stress. These synergies between plants, soil microbes, and metabolites provided an important protective mechanism for plant growth under adverse conditions.

## 5 Conclusion

This study revealed the mechanism through which sorghum-peanut intercropping significantly altered the microbial community structure of sorghum under salt stress by influencing soil sugar metabolism pathways. Under salt stress, sorghum-peanut intercropping significantly altered the metabolic profiles of the sorghum rhizosphere soil, particularly enhancing the consumption of sugar metabolites (fructose and sucrose) by influencing the sugar metabolic pathway, which provided essential energy support for plants and microbes to withstand the salt stress environment. Meanwhile, salt stress significantly changed the structure of the rhizosphere microbial community, while intercropping further optimized the structure of the microbial community by increasing specific beneficial microbes (*Rhodanobacter* and *Rhizopus*). Correlation analysis revealed that these microbial taxa exhibited significant correlations with sugar metabolites, implying that changes in metabolites directly affected the composition and functionality of microbial communities. Sorghum-peanut intercropping under salt stress optimized the rhizosphere microbial community structure of sorghum by modulating soil sugar metabolism pathways, thereby potentially improving the adaptability of sorghum under salt stress. The results from this study provide a potential new strategy for the sustainable use of salinized land.

## Data Availability

The datasets presented in this study can be found in online repositories. The names of the repository/repositories and accession number(s) can be found at: https://www.ncbi.nlm.nih.gov/, PRJNA1234366.
