# Supplementary material for: Sorghum-peanut intercropping under salt stress mediates rhizosphere microbial community shaping in sorghum by affecting soil sugar metabolism pathways
Source: Front Microbiol. 2025 May 1;16:1589415. doi: 10.3389/fmicb.2025.1589415 (PMC12078205; doi:10.3389/fmicb.2025.1589415)
Supplement: Supplementary file 1 [file Table_1.docx]

Supplementary Material


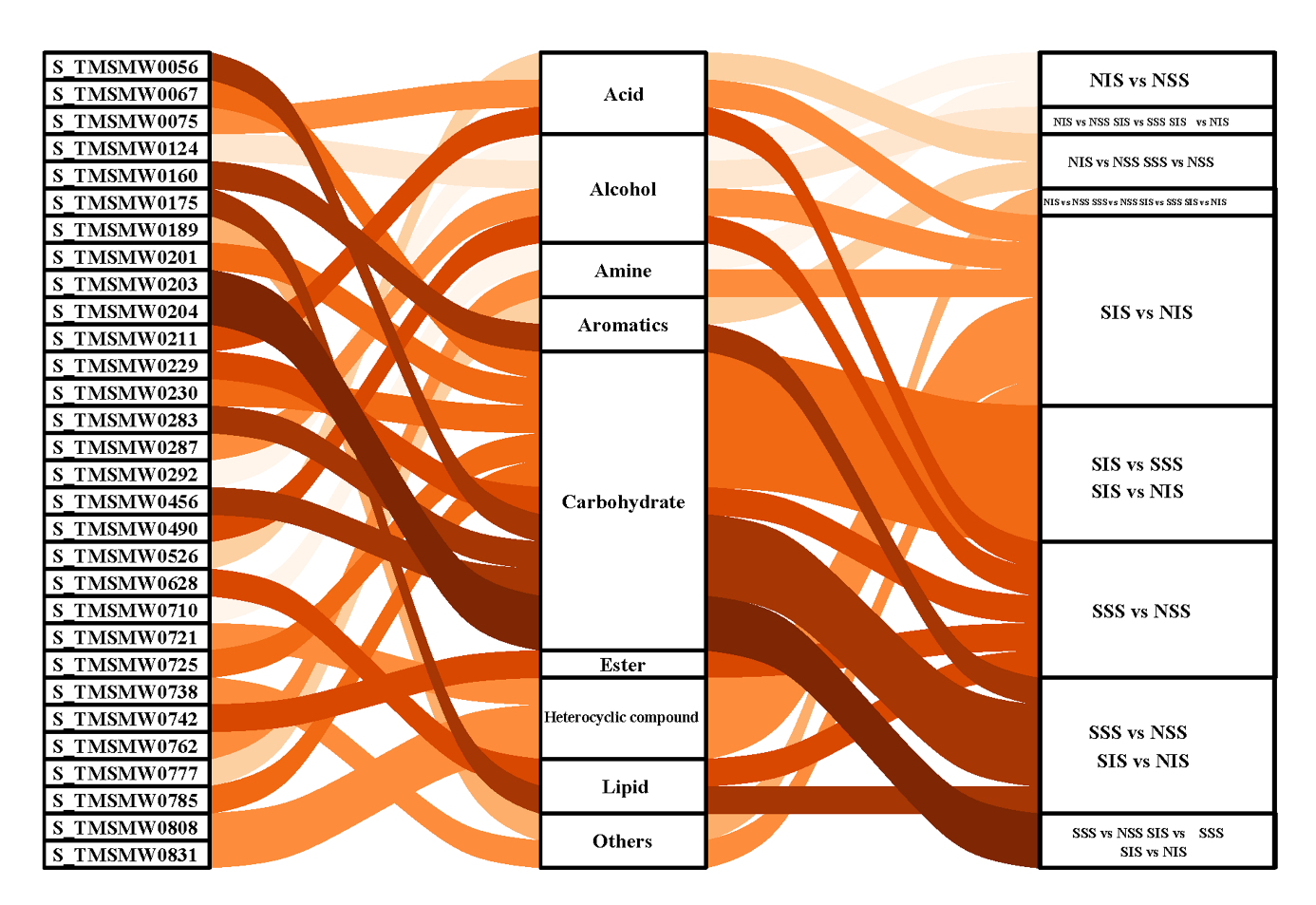


**Supplementary Figure 1.** Differential expressed metabolite classification Sankey diagram.

**
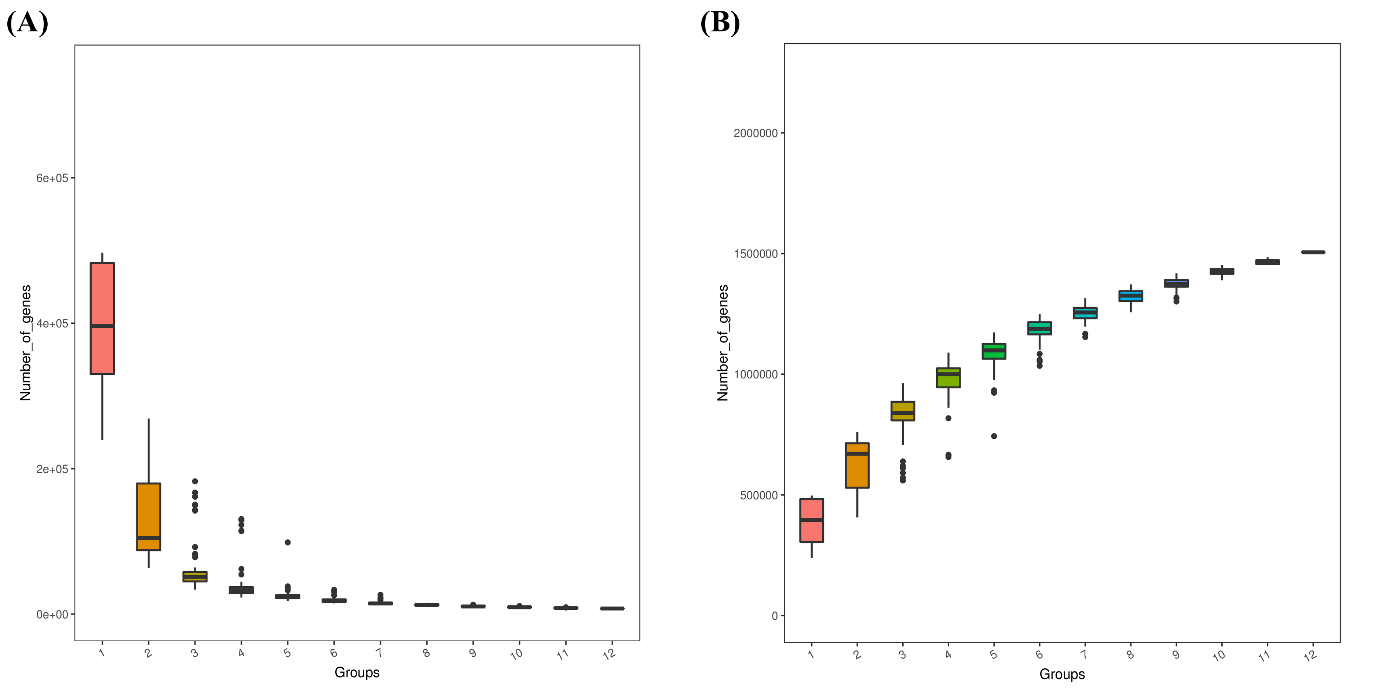
**

**Supplementary Figure 2.** Core-pan genetic analysis.


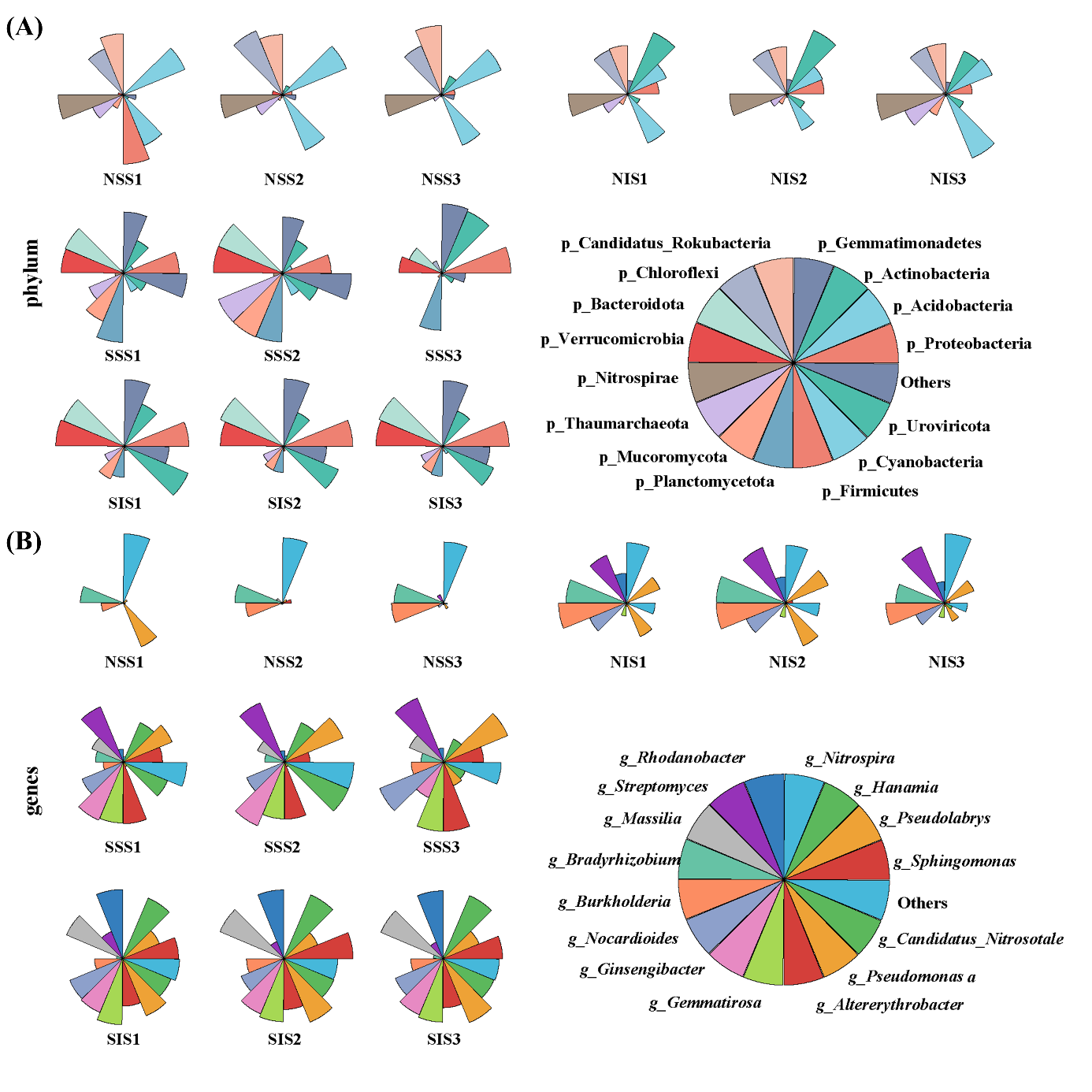


**Supplementary Figure 3.** Relative abundance of dominant microbial taxa in sorghum rhizosphere soil. (A–B) The relative abundance of dominant microbial taxa at the phylum and genus levels, respectively.

**Supplementary Table 1.** Soil physical and chemical properties of the test site

| Type | AHN | AP | AK | SOM | Soil soluble salt |
| --- | --- | --- | --- | --- | --- |
| Content | 96.70mg Kg^−1^ | 27.50mg Kg^−1^ | 117.9mg Kg^−1^ | 15.17g Kg^−1^ | 680mg Kg^−1^ |

AHN: alkali-hydrolyzable nitrogen; AP: available phosphorus; AK: available potassium; SOM: soil contained organic matter.

**Supplementary Table 2.** Rhizosphere soil metabolite composition of sorghum in all treatments

| Index | Formula | Compounds | Class I |
| --- | --- | --- | --- |
| S_TMSMW0011 | C_3_H_5_N_3_O_2_ | 4-Methylurazole | Others |
| S_TMSMW0012 | C_6_H_11_NO | 1-acetyl-2-methyl-Azetidine | Others |
| S_TMSMW0013 | CH_2_N_2_ | Methanediimine | Amine |
| S_TMSMW0014 | C_4_H_9_NO | N,N-Dimethylacetamide | Amine |
| S_TMSMW0018 | C_11_H_24_ | 4-methyl-Decane | Lipid |
| S_TMSMW0028 | C_3_H_7_ClO_2_ | 3-chloro-1,2-Propanediol | Alcohol |
| S_TMSMW0030 | C_12_H_26_ | Dodecane | Lipid |
| S_TMSMW0056 | C_5_H_10_O_5_ | d-Arabinose 2 | Carbohydrate |
| S_TMSMW0067 | C_6_H_12_O_6_ | D-Allose 1 | Carbohydrate |
| S_TMSMW0075 | C_17_H_34_O_2_ | Heptadecanoic Acid | Acid |
| S_TMSMW0077 | C_18_H_34_O_2_ | cis-Vaccenic Acid | Acid |
| S_TMSMW0112 | CH_2_O_3_ | carbonic Acid 1 | Acid |
| S_TMSMW0114 | C_7_H_7_FO_2_ | 4-Fluoro-2-methoxyPhenol | Phenol |
| S_TMSMW0123 | BH_3_O_3_ | Boric Acid | Acid |
| S_TMSMW0124 | C_3_H_8_O_2_ | Propylene Glycol | Alcohol |
| S_TMSMW0132 | C_8_H_11_NO_2_ | DopAmine | Amine |
| S_TMSMW0134 | C_3_H_6_O_3_ | Lactic Acid | Acid |
| S_TMSMW0137 | C_6_H_12_O_2_ | Hexanoic Acid 1 | Acid |
| S_TMSMW0140 | C_8_H_18_O | 2-ethyl-1-Hexanol | Alcohol |
| S_TMSMW0141 | C_13_H_28_ | 5-methyl-Dodecane | Lipid |
| S_TMSMW0144 | CH_4_N_2_O_2_ | Hydroxyurea | Nitrogen compounds |
| S_TMSMW0150 | C_15_H_34_OP_2_ | 1-(diisopropylphosphino)-3-(diisopropylphosphinyl)-Propane | Others |
| S_TMSMW0151 | C_3_H_4_O_4_ | Propanedioic Acid 2 | Acid |
| S_TMSMW0152 | C_13_H_28_ | 2,7-dimethyl-Undecane | Lipid |
| S_TMSMW0160 | C_16_H_18_O_2_ | 2-(4'-Hydroxyphenyl)-2-(4'-methoxyphenyl)propane | Aromatics |
| S_TMSMW0161 | C_3_H_8_O_3_ | Glycerin | Alcohol |
| S_TMSMW0162 | C_13_H_28_ | 4,7-dimethyl-Undecane | Lipid |
| S_TMSMW0165 | C_8_H_18_O_3_ | 2-(2-butoxyethoxy)-Ethanol | Alcohol |
| S_TMSMW0167 | C_9_H_18_O_2_ | Nonanoic Acid | Acid |
| S_TMSMW0175 | C_12_H_25_I | 1-iodo-Dodecane | Lipid |
| S_TMSMW0176 | C_14_H_22_O | 2,4-Di-tert-butylPhenol 2 | Phenol |
| S_TMSMW0179 | CH_3_N_5_ | 5H-Tetrazol-5-amine | Others |
| S_TMSMW0182 | C_11_H_24_ | 2,4,6-trimethyl-Octane | Lipid |
| S_TMSMW0189 | C_18_H_22_O_2_ | 2-(4'-Methoxyphenyl)-2-(3'-methyl-4'methoxyphenyl)propane | Others |
| S_TMSMW0199 | C_14_H_28_O_2_ | Tetradecanoic Acid | Acid |
| S_TMSMW0201 | C_6_H_12_O_6_ | Fructose 2 | Carbohydrate |
| S_TMSMW0203 | C_6_H_12_O_6_ | D(+)-Talose | Carbohydrate |
| S_TMSMW0204 | C_6_H_12_O_6_ | D-Allose 2 | Carbohydrate |
| S_TMSMW0211 | C_16_H_32_O_2_ | n-Hexadecanoic Acid | Acid |
| S_TMSMW0219 | C_18_H_36_O_2_ | Octadecanoic Acid | Acid |
| S_TMSMW0220 | C_30_H_52_F_3_N | 3-Trifluoromethylbenzylamine, N,N-diundecyl | Others |
| S_TMSMW0225 | C_19_H_38_O_4_ | Glycerol 1-palmitate | Ester |
| S_TMSMW0229 | C_5_H_10_O_5_ | D-(-)-Ribofuranose 1 | Carbohydrate |
| S_TMSMW0230 | C_12_H_22_O_11_ | Sucrose | Carbohydrate |
| S_TMSMW0234 | C_21_H_42_O_4_ | Octadecanoic acid, 2,3-dihydroxypropyl Ester | Ester |
| S_TMSMW0235 | C_22_H_43_NO | (Z)-13-Docosenamide | Amine |
| S_TMSMW0237 | C_8_H_16_O_6_ | Ethyl .alpha.-d-glucopyranoside | Carbohydrate |
| S_TMSMW0261 | CH_3_NO_2_ | carbamic Acid 2 | Acid |
| S_TMSMW0262 | C_4_H_10_O_2_ | 1,3-Butanediol | Alcohol |
| S_TMSMW0265 | C_7_H_8_O | Benzyl Alcohol | Alcohol |
| S_TMSMW0277 | C_15_H_32_ | 2,7,10-trimethyl-Dodecane | Lipid |
| S_TMSMW0283 | C_6_H_10_O_5_ | 1,6-anhydro-.beta.-D-Glucopyranose | Carbohydrate |
| S_TMSMW0287 | C_7_H_14_O_6_ | D-Pinitol | Alcohol |
| S_TMSMW0292 | C_6_H_12_O_6_ | Myo-Inositol 2 | Alcohol |
| S_TMSMW0310 | C_29_H_50_O | .beta.-Sitosterol | Alcohol |
| S_TMSMW0350 | C_17_H_36_ | Heptadecane | Lipid |
| S_TMSMW0354 | C_6_H_14_O_3_ | 2-ethyl-2-(hydroxymethyl)-1,3-Propanediol | Alcohol |
| S_TMSMW0355 | C_14_H_30_ | Tetradecane | Lipid |
| S_TMSMW0365 | C_16_H_34_ | Hexadecane | Lipid |
| S_TMSMW0383 | C_17_H_34_O_4_ | Tetradecanoic acid, 2,3-dihydroxypropyl Ester | Ester |
| S_TMSMW0384 | C_22_H_41_N | (Z)-Docos-9-enenitrile | Others |
| S_TMSMW0401 | C_5_H_11_NO | N,N-diethyl-Formamide | Amine |
| S_TMSMW0415 | C_5_H_5_NO | 4-Pyridinol | Heterocyclic compound |
| S_TMSMW0418 | C_12_H_26_ | 4-methyl-Undecane | Lipid |
| S_TMSMW0431 | C_4_H_10_O_3_ | 2,2'-oxybis-Ethanol | Alcohol |
| S_TMSMW0443 | C_12_H_26_ | 2-methyl-Undecane | Lipid |
| S_TMSMW0448 | C_12_H_26_ | 2,9-dimethyl-Decane | Lipid |
| S_TMSMW0456 | C_6_H_12_O_5_ | Rhamnose | Carbohydrate |
| S_TMSMW0488 | C_9_H_13_NO_3_ | Epinephrine | Amine |
| S_TMSMW0490 | C_29_H_48_O | Stigmasterol 2 | Alcohol |
| S_TMSMW0495 | C_19_H_38_O_4_ | Hexadecanoic acid, 2-hydroxy-1-(hydroxymethyl)ethyl Ester | Ester |
| S_TMSMW0496 | C_24_H_38_O_4_ | Phthalic acid, di(oct-3-yl) ester | Lipid |
| S_TMSMW0504 | C_11_H_24_ | 2,6,6-trimethyl-Octane | Lipid |
| S_TMSMW0526 | C_16_H_30_O_2_ | 9-Hexadecenoic Acid | Acid |
| S_TMSMW0530 | C_14_H_30_ | 4,6-dimethyl-Dodecane | Lipid |
| S_TMSMW0532 | F_2_H_2_NP | Phosphoramidous difluoride | Others |
| S_TMSMW0544 | C_15_H_12_ | 1,1'-(1,2-propadienylidene)bis-Benzene | Aromatics |
| S_TMSMW0564 | C_8_H_11_NO | (R)-(-)-2-Phenylglycinol | Alcohol |
| S_TMSMW0588 | C_6_H_14_O_6_ | Sorbitol 1 | Carbohydrate |
| S_TMSMW0589 | C_22_H_27_NO_4_ | 6-(4-ethoxyphenyl)-3-methyl-4-oxo-4,5,6,7-tetrahydro-1H-Indole-2-carboxylic acid, isobutyl ester | Heterocyclic compound |
| S_TMSMW0602 | C_8_H_10_O4 | dl-3,4-Dihydroxyphenylglycol | Alcohol |
| S_TMSMW0604 | C_11_H_23_I | 1-Iodoundecane | Others |
| S_TMSMW0613 | C_13_H_28_ | 6-ethyl-2-methyl-Decane | Lipid |
| S_TMSMW0621 | C_13_H_17_NO | 4-ethyl-N-methallyl-Benzamide | Others |
| S_TMSMW0626 | C_9_H_13_NO | (.+/-.)-Norephedrine | Amine |
| S_TMSMW0628 | C_18_H_28_O_3_ | methyl 3-(3,5-ditert-butyl-4-hydroxyphenyl)propanoate | Lipid |
| S_TMSMW0664 | C_13_H_28_ | 4,6-dimethyl-Undecane | Lipid |
| S_TMSMW0669 | C_11_H_24_ | 2,5-dimethyl-Nonane | Lipid |
| S_TMSMW0670 | C_9_H_20_ | 4-methyl-Octane | Lipid |
| S_TMSMW0679 | C_11_H_24_ | 4,5-dimethyl-Nonane | Lipid |
| S_TMSMW0693 | C_13_H_28_ | 4,8-dimethyl-Undecane | Lipid |
| S_TMSMW0701 | C_14_H_22_O_2_ | 4,6-di-tert-Butylresorcinol | Phenol |
| S_TMSMW0708 | C_19_H_40_ | Nonadecane | Lipid |
| S_TMSMW0709 | C_15_H_32_ | 4-methyl-Tetradecane | Others |
| S_TMSMW0710 | C_5_H_14_N_2_ | 1,5-PentanediAmine | Amine |
| S_TMSMW0714 | C_6_H_14_BNO | 2-butyl-1,3,2-Oxazaborolane | Heterocyclic compound |
| S_TMSMW0718 | C_14_H_22_O | 2,4-Di-tert-butylPhenol 1 | Phenol |
| S_TMSMW0721 | C_4_H_8_O_2_S_2_ | trans-O-Dithiane-4,5-diol | Heterocyclic compound |
| S_TMSMW0725 | C_5_H_12_O_4_ | 1-Deoxy-d-arabitol | Carbohydrate |
| S_TMSMW0736 | C_21_H_44_ | Heneicosane | Lipid |
| S_TMSMW0737 | C_4_H_7_N_5_O | N-(2-Methyl-2H-tetrazol-5-yl)-acetamide | Heterocyclic compound |
| S_TMSMW0738 | C_20_H_41_I | 1-iodo-Eicosane | Others |
| S_TMSMW0740 | C_21_H_32_F_3_NO | N-trifluoroacetyl-2,4,6-Tri-tert-butyl-N-methylaniline | Amine |
| S_TMSMW0742 | C_20_H_30_O5 | Andrographolide | Ester |
| S_TMSMW0747 | C_25_H_39_NP_2_ | [2-(6-methyl-2-pyridyl)ethyl](phenyl)-[3-(di-t-butylphosphino)propyl]-Phosphine | Heterocyclic compound |
| S_TMSMW0760 | C_5_H_10_N_2_O | 2-amino-N-cyclopropylacetamide | Amine |
| S_TMSMW0762 | C_18_H_35_NO | (Z)-9-Octadecenamide 1 | Amine |
| S_TMSMW0763 | C_18_H_35_NO | (Z)-9-Octadecenamide 2 | Amine |
| S_TMSMW0764 | C_18_H_37_NO | Octadecanamide | Amine |
| S_TMSMW0777 | C_7_H_5_NO | 4-hydroxy-Benzonitrile | Aromatics |
| S_TMSMW0785 | C_6_H_12_O_6_ | Fructose 1 | Carbohydrate |
| S_TMSMW0790 | C_17_H_30_O_12_ | 4-(2-Methylbutanoyl)Sucrose | Carbohydrate |
| S_TMSMW0808 | C_11_H_10_O_5_ | 1,3-Benzodioxole-5-(4-keto-butyric acid) | Heterocyclic compound |
| S_TMSMW0829 | C_20_H_28_CoN_2_ | bis(.eta.-5-piperidinylcyclopentadienyl)-Cobalt | Heterocyclic compound |
| S_TMSMW0831 | C_18_H_22_N_2_O_2_ | 5,6,7,8-tetrahydro-2-amino-4-(3-cyclohexenyl)-7,7-dimethyl-5-oxo-4H-Benzo[b]pyrane-3-carbonitrile | Heterocyclic compound |
| S_TMSMW0841 | C_9_H_20_ | 4-ethyl-Heptane | Lipid |
| S_TMSMW0843 | C_12_H_22_O_4_ | (1R,2S,5R)-2-isopropyl-5-methylcyclohexyl 2,2-dihydroxyacetate | Ester |
| S_TMSMW0844 | C_13_H_28_ | 2,6-dimethyl-Undecane | Lipid |
| S_TMSMW0850 | C_14_H_30_ | 2,3-Dimethyldodecane | Lipid |
| S_TMSMW0856 | C_15_H_32_ | 3-methyl-Tetradecane | Lipid |
| S_TMSMW0857 | C_5_H_10_O_5_ | (3S,4R)-3,4,5-trihydroxypentanoic acid | Carbohydrate |
| S_TMSMW0859 | C_14_H_24_O_5_ | Succinic acid, 3-methylbut-2-yl tetrahydrofurfuryl ester | Lipid |
| TMS_IS_08 | 13C_5_H_12_O_5_ | Ribitol-13C5 | carbohydrate |
